# Current trends and future directions in probiotics research for HIV/AIDS

**DOI:** 10.3389/fmicb.2024.1444552

**Published:** 2024-12-27

**Authors:** Xinxin Cui, Zhanpeng Xie, Zhen Wu, Li-qin Xu

**Affiliations:** ^1^Medical School of Shihezi University, Xinjiang, China; ^2^Yueyang County People’s Hospital, Yueyang, China

**Keywords:** HIV/AIDS, probiotics, biblimetrics, citespace, VOSveiwer

## Abstract

**Aim:**

This study aims to comprehensively and systematically review the current status of research on probiotics and HIV/AIDS, while also exploring future research hotspots and trends in this domain.

**Methods:**

The Web of Science (WoS) Core Collection database was queried up until May 13, 2024, to retrieve relevant literature on probiotics and HIV/AIDS. Utilizing CiteSpace, VOSviewers, and Bibliometrix software, scientific achievements and research frontiers in this field were analyzed.

**Results:**

As of May 14, 2024, a total of 90 articles was included in. The publication output in this area peaked in 2017, with a subsequent decline in the number of articles post-2019. The United States emerged as the leading country in terms of article count (32 articles), with The University of Western Ontario being the institution with the highest publication output. Dr. Reid G contributed the most articles (12 articles). In addition to key terms, high-frequency keywords included immune activation, inflammation, and microbial translocation. The burst analysis of keywords suggests that vaccines may become a focal point of future research.

**Conclusion:**

Future research hotspots and trends should focus on elucidating the types of probiotics, intervention timing, and optimal strains (in terms of mixing ratios) in the context of HIV/AIDS. Furthermore, exploration into the role of probiotic metabolites, such as short-chain fatty acids, in vaccine development is warranted.

## 1 Introduction

Acquired Immunodeficiency Syndrome (AIDS) primarily stems from infection with the Human Immunodeficiency Virus (HIV) (Li et al., 2024). HIV predominantly targets CD4+ T cells (Meng et al., 2024). Highly Active Antiretroviral Therapy (HAART) is a therapeutic approach to treating AIDS by combining three or more antiretroviral drugs, which has significantly reduced the mortality rate of HIV-1-infected patients. It was a significant achievement of modern medicine (Jilek et al., 2012). Persons Living with HIV (PLWH) who had not undergone HAART experience progressive depletion of CD4+ T cells, leading to succumbing to various infections and tumors (Burkhart Colorado et al., 2024). Since the introduction of HAART in 1996 for the management of AIDS, the life expectancy of infected individuals has significantly improved. However, a study showed that approximately 10 to 40% of those infected exhibit characteristics of immunological non-responders (Yang et al., 2020). These individuals showed no elevation in CD4+ T cell counts despite HAART administration, and the chronic inflammation and sustained immune system activation in PLWH remain unresolved (Rhoades et al., 2019).

Chronic inflammation and sustained immune activation augment the risk of inflammatory non-AIDS events, such as cardiovascular diseases, among PLWH (Trøseid et al., 2024). Dysbiosis of the probiotics contributed significantly to chronic inflammation. It sustained immune system activation and also contributed to immunological non-response, with non-responders exhibiting higher mortality rates than responders (Lohani et al., 2024). *Akkermansia muciniphila*, by modulating mucin thickness, facilitated intestinal mucosal homeostasis. Enrichment of certain species within the Lachnospiraceae and Ruminococcaceae families induced the expansion of regulatory T cells, thereby dampening detrimental inflammatory responses (Vujkovic-Cvijin et al., 2020). Consequently, targeting the probiotics for the treatment of AIDS was imperative. Studies have indicated that dietary supplementation improved intestinal barrier function in individuals with AIDS while also promoting immune reconstitution (Lu et al., 2024).

Furthermore, research by Blázquez-Bondia et al. (2022) suggested that probiotic intervention was safe and led to a slight increase in the CD4/CD8 ratio, although its clinical significance remained uncertain. Hence, a comprehensive and systematic review of this field was essential to address these disparities and provide insights into future research hotspots and trends.

Bibliometric analysis allows summarizing existing publications in the field related to AIDS and probiotics, analyzing the structure of research and quantitative information in a particular research area. The relative contribution of different countries, authors and journals in this field can be provided in a visual map. Thus, bibliometric analysis can outline the current general framework and show the focus and trends in the field (Gu et al., 2021). This study employed Citespace.6.1.R6, VOSviewers1.6.19, and Bibliometrix to analyze literature published before May 13, 2024, concerning “AIDS and probiotics,” thereby elucidating changes in hotspots and trends to guide future research directions. There have been no reports on bibliometric studies regarding probiotics and AIDS.

## 2 Materials and methods

### 2.1 Database source

The Web of Science (WOS) Core Collection was selected for retrieval, encompassing literature published from the database’s inception until May 13, 2024.

### 2.2 Search strategy

This study employed Medical Subject Headings (MeSHs) terms from the PubMed database to maximize article coverage. The search strategy was tailored according to Web of Science, focusing on HIV/AIDS AND probiotics and associated terminology (refer to the Supplementary materials for specific search tactics).

### 2.3 Literature selection and inclusion criteria

Only research articles were included in this study, with searches restricted to English-language publications. Two researchers independently conducted the screening. A total of 530 records were retrieved, and manual filtering was performed based on the relevance of titles, abstracts, and keywords to “probiotics and AIDS.” Full-text assessment was conducted when insufficient information was available for determination. After deduplicating and excluding clearly ineligible articles, the two researchers retrieved 75 and 104 articles, respectively. After the third researcher judged the divergence between the two, 90 articles were included. The study workflow was depicted in Supplementary Figure S1.

### 2.4 Analytical methods

The 90 articles were exported and downloaded in Plain Text File, Tab Delimited File, and BibTeX formats. CiteSpace (6.1.R6) was utilized for burst keyword analysis, journal dual-map overlay, and keyword timeline visualization, with co-occurrence network construction. VOSviewers (1.6.18) were employed to identify countries, institutions, journals, and keywords and generate co-occurrence networks. Bibliometrix in RStudio version 4.2.3 facilitated visual analysis of countries and authors. Microsoft Excel (2019) was utilized for data management and analysis of annual publications and citation trends.

Ethical approval was not applicable for the present study.

## 3 Results

### 3.1 Publication status and journals

**Figure 1:**
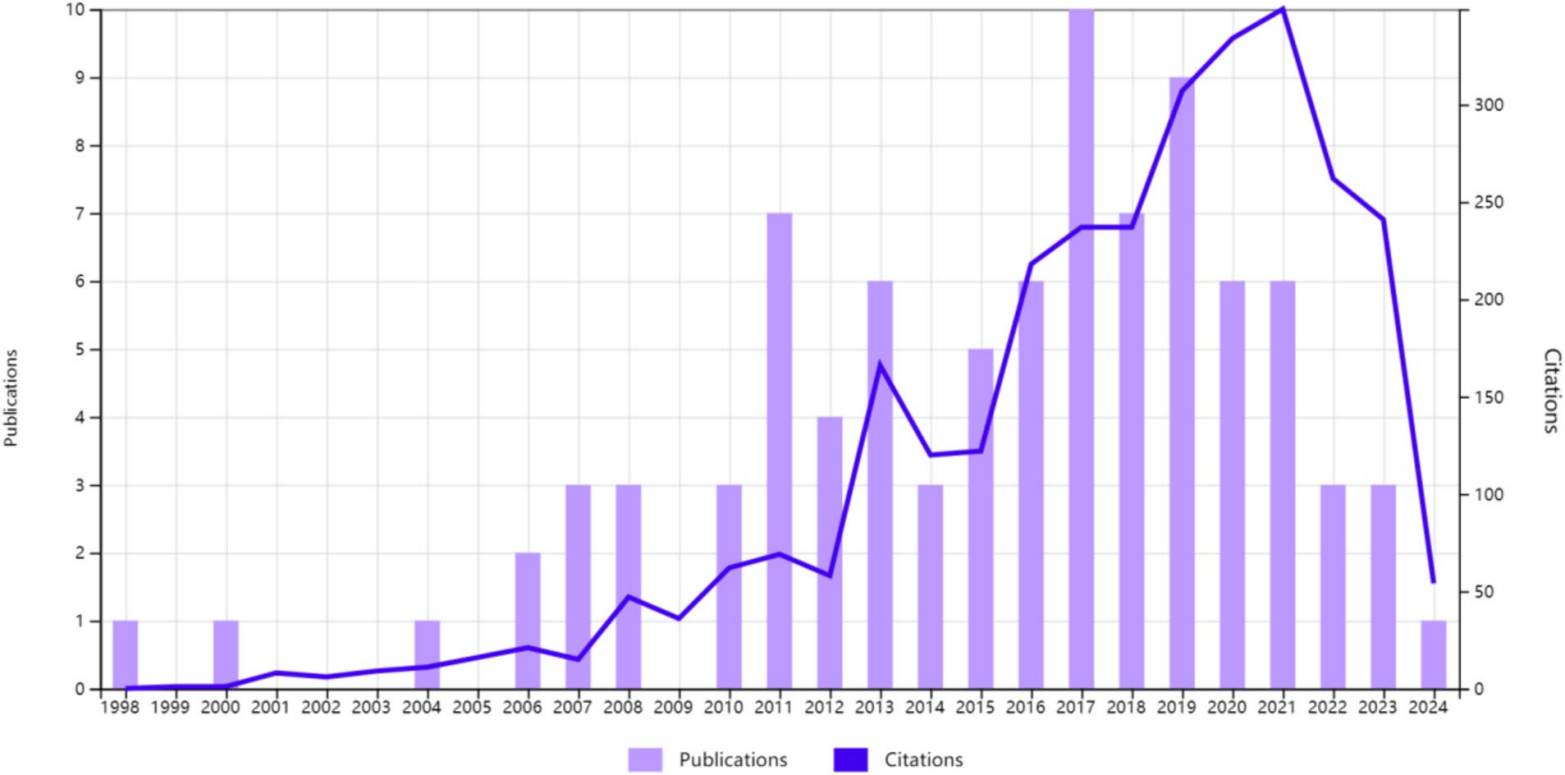
The volume of publications and citations fluctuates with the passage of time.

Ninety articles were included based on the search strategy and screening criteria. The production and citation counts of articles from January 1, 1998, to May 13, 2024, are depicted in Figure 1. Overall, the volume of publications was relatively low, with a peak of 10 articles in 2017. Articles on the intersection of probiotics and HIV/AIDS were published in 58 journals, as detailed in Supplementary Figure S2. The top 10 journals were presented in Supplementary Table S1. The journal with the highest impact factor was *GUT MICROBES* (IF: 12.2), while the most prolific journal was *Nutrients* (IF: 4.8), with six related articles published. Both of these journals are Journal Citation Reports (JCR) Q1. This suggested promising research prospects in this field, with potential for publication in high-quality journals.

**Figure 2:**
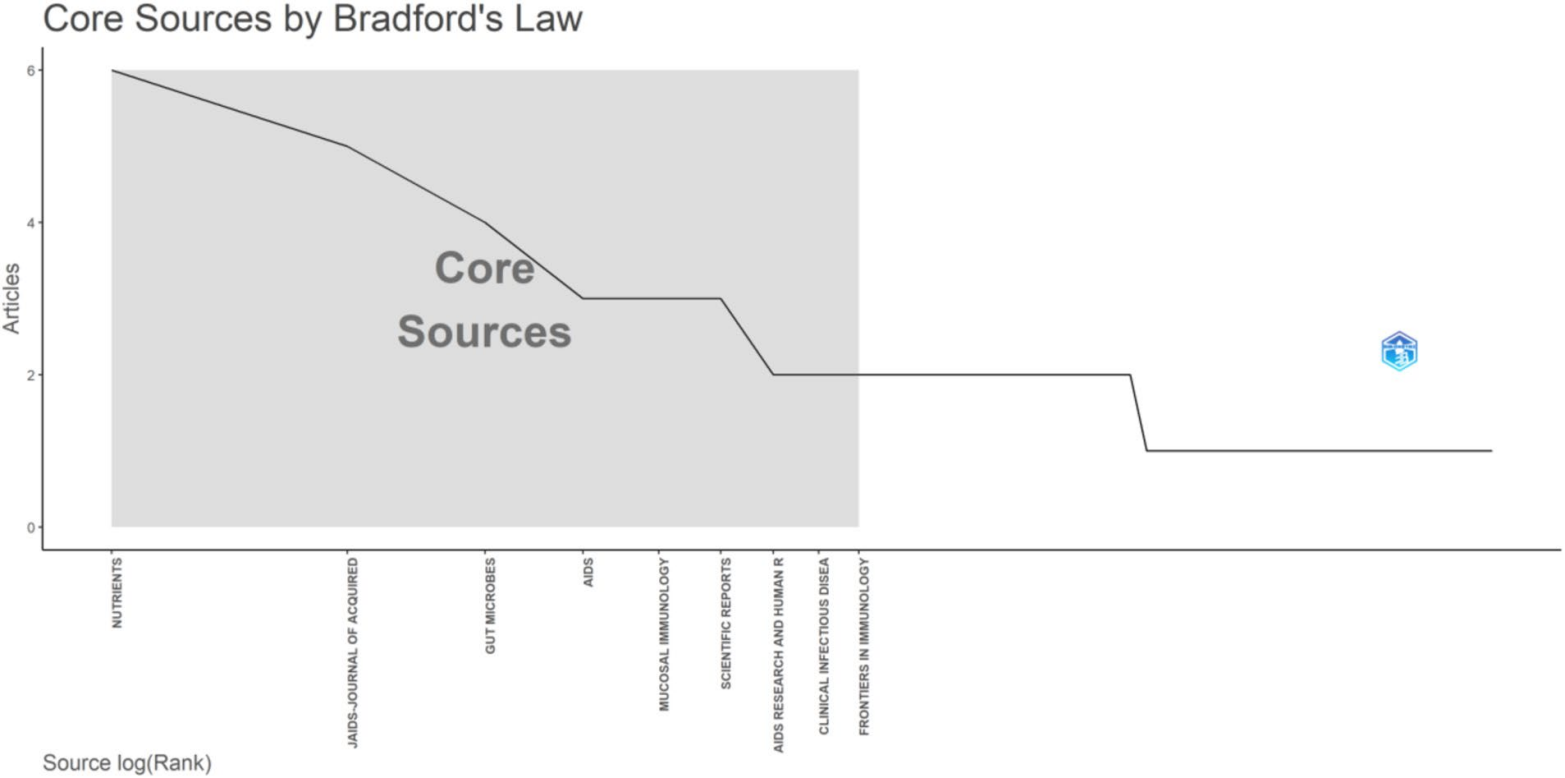
Core and non core journals.

**Figure 3:**
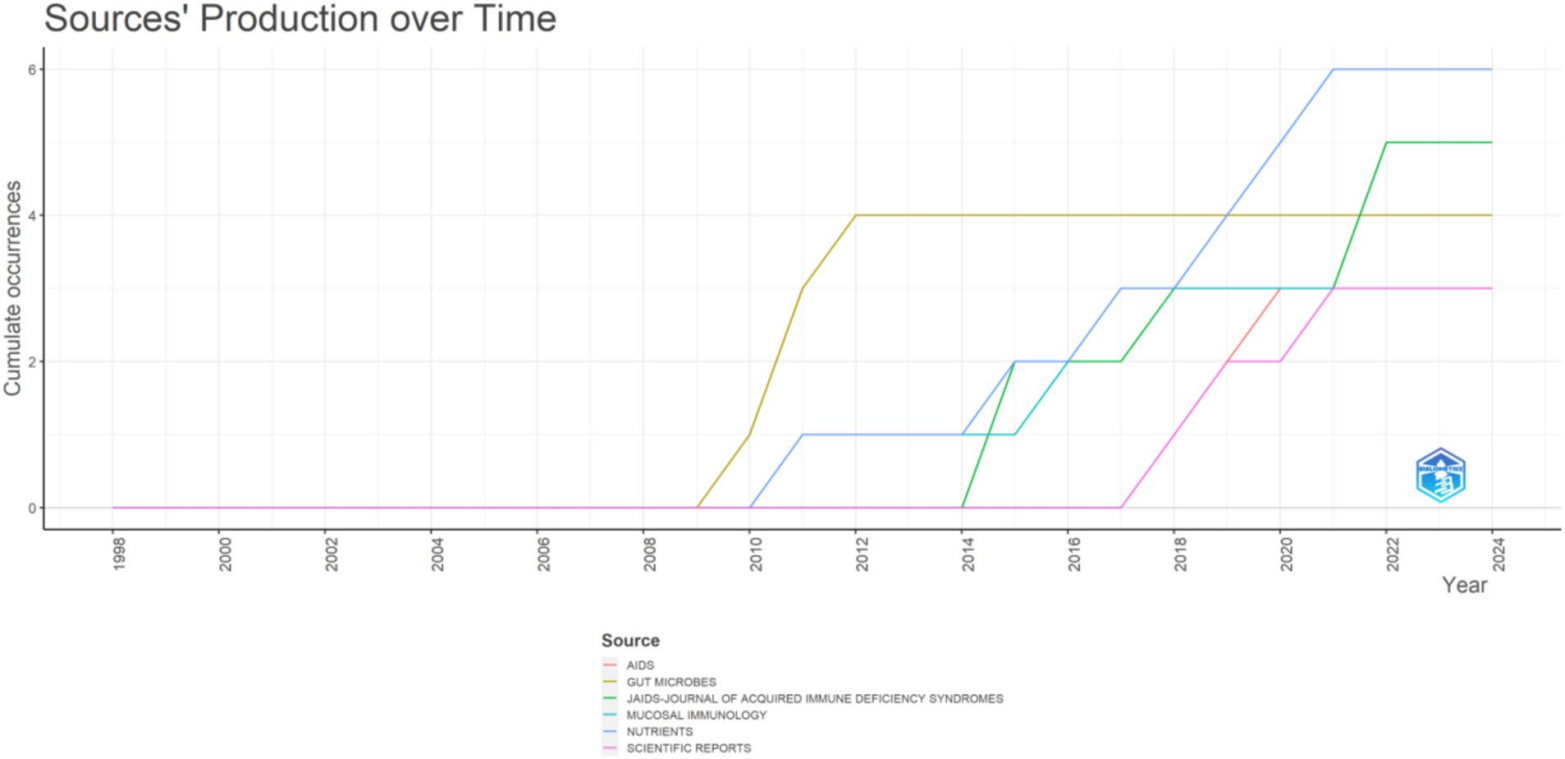
Journal publication volume between different years.

Furthermore, according to Bradford’s Law, these journals were considered core journals. However, over time, publications in these journals may have plateaued (see Figures 2, 3). Additionally, a journal overlay analysis was conducted for AIDS and probiotics (Supplementary Figure S3) to explore further the thematic distribution of journals and the transfer pathway of disciplinary knowledge. The citing journals predominantly included clinical, molecular biology, medical, immunology, and clinical journals, while the cited journals mainly comprise molecular, biological, and genetic journals.

### 3.2 Visualization analysis of authors, countries, and institutions collaboration

**Figure 4:**
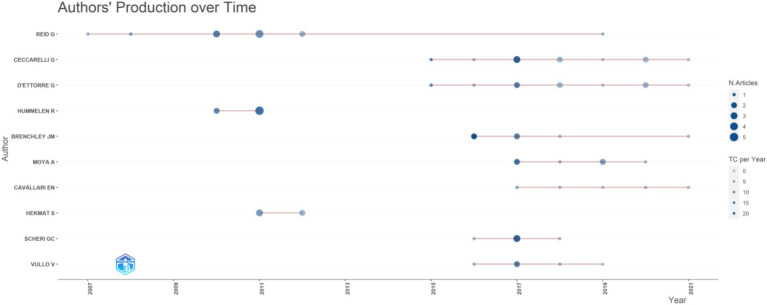
The quantity of articles written by the author fluctuates over time.

**Figure 5:**
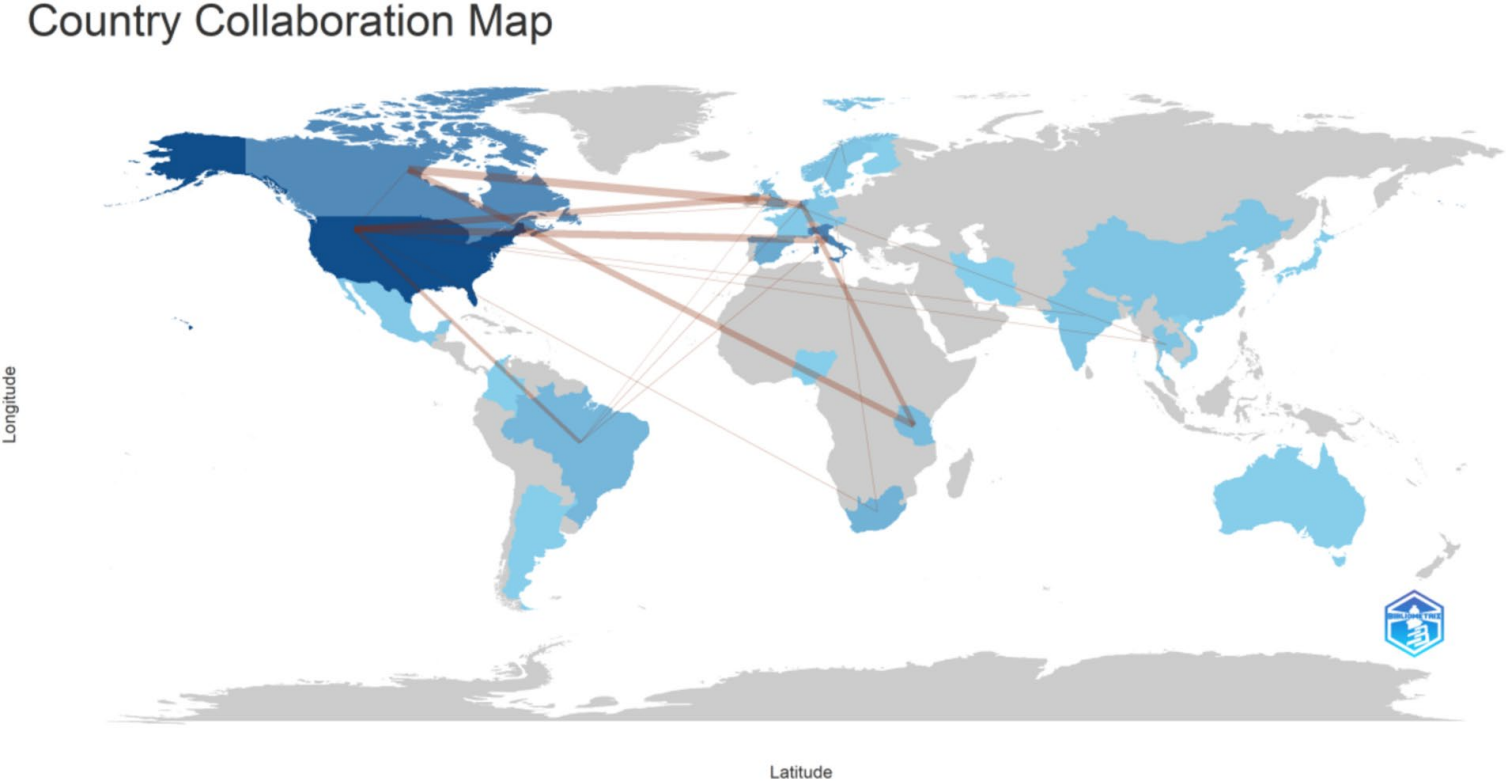
The visualization of national output collaboration. With darker colors representing higher output and thicker lines indicating closer collaboration.

**Figure 6:**
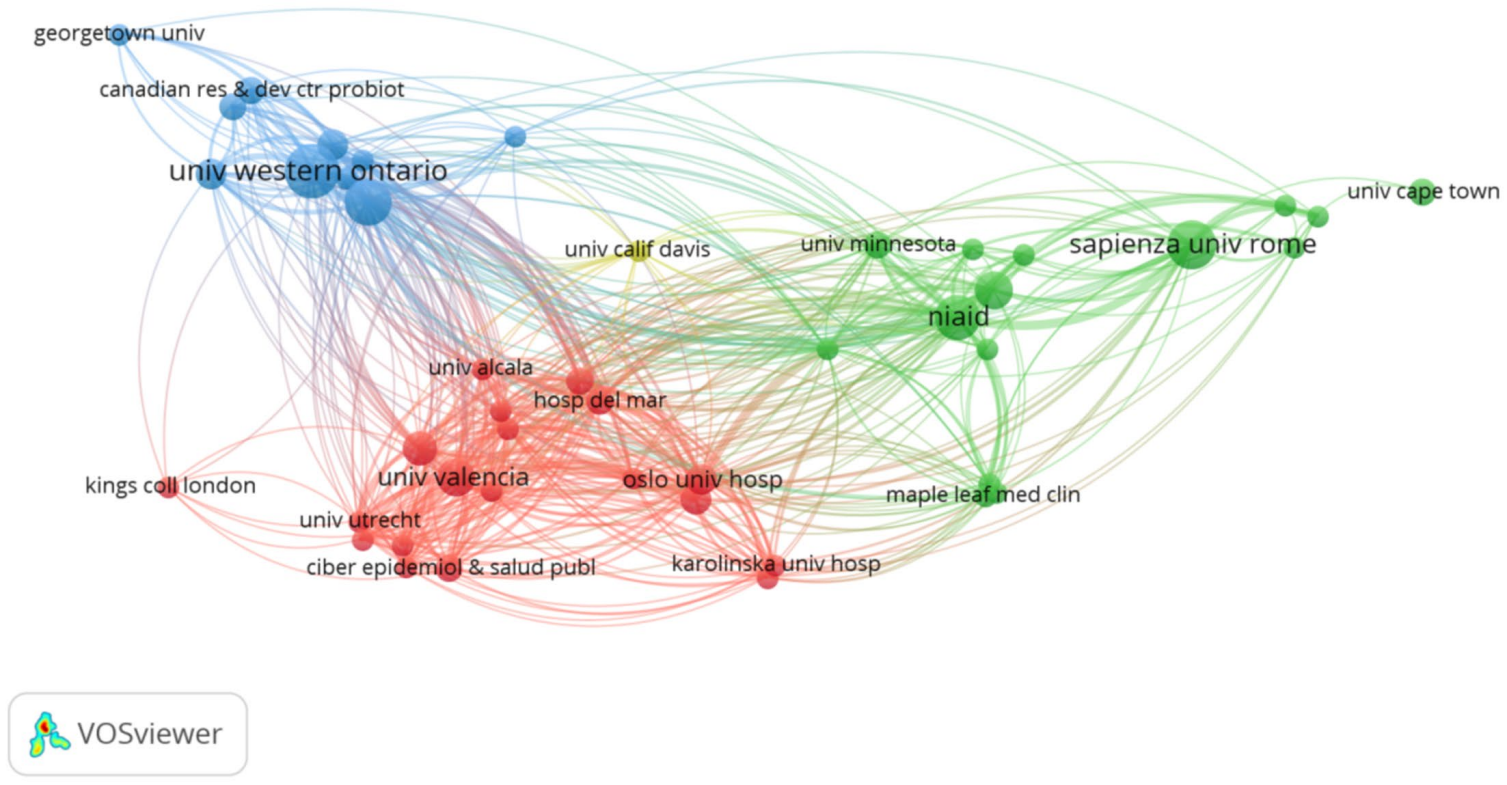
The institution produces visual analyses. Where the size of the nodes represents the volume of publications, and the thickness of the connections represents the level of collaboration.

The top 10 authors by publication count were depicted in Figure 4, with Reid G ranking first with 12 publications. Reid G’s research primarily focused on *Lactobacillus rhamnosus GR-1* and *Lactobacillus reuteri RC-14*, which target female subjects (Liu et al., 2007; Anukam et al., 2008; Irvine et al., 2010; Hummelen et al., 2010; Hummelen et al., 2011a; Hemsworth et al., 2011; Dols et al., 2011; Hummelen et al., 2011b; Dizzell et al., 2019). A total of 28 countries were involved in research on AIDS and probiotics, as visualized in Figure 5. The United States published 32 articles, followed by Italy with 18 articles. Three countries have published more than 10 articles each. Collaboration among countries was predominantly led by high-output nations, with exchanges occurring between various nations. A total of 248 institutions have contributed to publications. The University -of Western Ontario ranked first in publication count with 12 articles, and the top 10 institutions were all from developed countries (Figure 6). The institution burst map was provided in Supplementary Figure S4, highlighting institutions such as George Washington University, University of Washington, and University of Minnesota, all from the United States, indicating a potential for increased publication output from the United States in this field in the future.

### 3.3 Analysis of articles’ citations

The top 10 cited articles were presented in Supplementary Table S2. These studies have relatively small sample sizes, not exceeding a hundred (Irvine et al., 2010; Hummelen et al., 2011a; Villar-García et al., 2015; Cunningham-Rundles et al., 2000; D’Ettorre et al., 2015; Wolf et al., 1998; Anukam et al., 2008; Mudd and Brenchley, 2016; Klatt et al., 2013; Gori et al., 2011). They suggested that probiotic supplementation can reduce inflammation and immune activation and alleviate microbial translocation, but the results regarding the increase in CD4+ T cell count were inconsistent. While some studies indicated a significant increase in CD4+ T cell counts with probiotic supplementation, others showed only a slight improvement. No burst articles were identified (Supplementary Figure S5), likely due to the limited number of publications. Additionally, review articles were excluded, which may have influenced this result.

### 3.4 Keyword analysis

**Figure 7:**
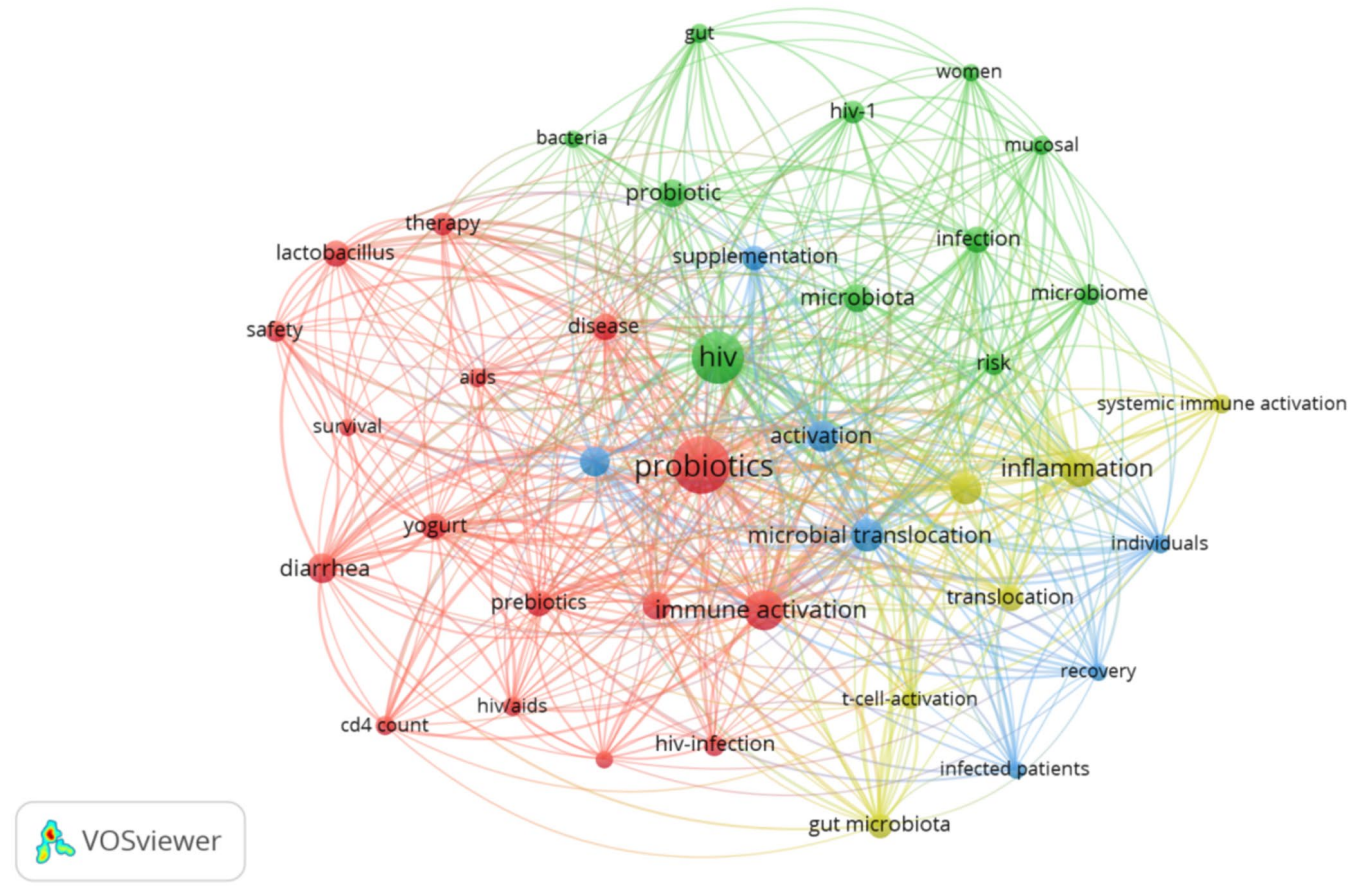
Keyword visual analysis. Node size represents keyword frequency, thickness represents the strength of connection.

A total of 546 keywords were included, forming a keyword map (Figure 7). Apart from the main terms, probiotics, HIV, immune activation, inflammation, and microbial translocation were the most frequently occurring keywords, all closely interrelated. The 20 most vital burst keywords are shown in Supplementary Figure S6, with “vaccine” appearing in 2023 and continuing to this day.

## 4 Discussion

### 4.1 Global publication and journal trends

The fluctuation in publication volume served as a crucial indicator of developmental trends. Our systematic review of articles on probiotic intervention in HIV/AIDS from 1998 to 2024 reveals peaks in publications in 2017 and 2019, with 10 and 9 articles, respectively. The first meta-analysis was published in 2021, a year in which publications in this field continued to decline, which may have affected publication volume.

The decline in publication volume may be attributed to three main factors: ① inconsistencies in studies regarding whether probiotics can reduce inflammation and increase CD4+ T cell count; ② the negative results of the first meta-analysis on probiotics’ effect on CD4+ T cell count in 2021; ③ our study did not include review articles in this field.

Numerous studies indicated an increase in pro-inflammatory bacteria (*Enterobacteriaceae* and *Desulfovibrionaceae*) and a decrease in anti-inflammatory bacteria (*Lachnospiraceae*, *Ruminococcaceae*, *Rikenellaceae*, and *Bacteroides*) in PLWH (Vujkovic-Cvijin et al., 2020). HIV also damaged the intestinal mucosal barrier, significantly increasing intestinal fatty acid-binding protein (IFABP) (MacCann et al., 2023). The above results lead to LPS leakage, further exacerbating inflammatory responses and sustained immune activation.

d’Ettorre et al. demonstrated a significant reduction in high-sensitivity C-reactive protein (hsCRP) after the probiotic intervention, a marker associated with a significant decrease in cardiovascular disease risk (D’Ettorre et al., 2015). A considerable number of Randomized Controlled Trial (RCT)studies suggested that probiotic supplementation played a significant role in slowing inflammation and immune activation and may also affect CD4+ T cell count (Mortezazadeh et al., 2023; Reale et al., 2021; Jones et al., 2021), although some studies showed that probiotic supplementation does not significantly impact CD4+ cell counts (Blázquez-Bondia et al., 2022; Rousseau et al., 2022; Meyer-Myklestad et al., 2022). Moreover, these meta-analyses did not classify probiotics. These differences need to be explored and added to by subsequent researchers.

Subgroup analyses on intervention duration revealed that only sufficient intervention time can reduce the incidence of AIDS-related diarrhea. The incidence of AIDS-related diarrhea decreased when probiotics were given for more than 30 days. When probiotics were given for less than 30 days, there was no change in the incidence of AIDS-related diarrhea (Kazemi et al., 2018). These studies suggested that probiotic treatment of AIDS-related diarrhea requires more than a 30-day course of therapy in clinical practice for the management of AIDS. Currently, there are meta-analyses regarding probiotic intervention in AIDS, with inconclusive results (Sachdeva et al., 2022; Zhang et al., 2021). Some argue that probiotics increased CD4+ T cell counts (children) in PLWH, while others contend that probiotics did not affect restoring CD4+ T cell counts. Future research should explore the optimal probiotic strains (mixed ratios), dosages, and administration timing to clarify their specific roles.

Through visualization analysis of journals, it was found that the first journal in this field was Nutrients, followed by *JAIDS-JOURNAL OF ACQUIRED IMMUNE DEFICIENCY SYNDROMES*, with the highest impact factor being *Gut Microbes*. Future attention should be focused on these journals to obtain cutting-edge information.

### 4.2 Countries, institutions, and authors

Regarding the analysis of countries, institutions, and authors, developed countries have far exceeded developing countries in terms of publication volume. Although the number of AIDS patients in developing countries was much higher than in developed countries, with two-thirds of AIDS patients in sub-Saharan Africa, developed countries have more abundant funds and research technology. Additionally, the top 10 institutions by publication count were all from developed countries, most of which are universities, indicating that research in this field requires strong research capabilities. Through analysis of the first authors of publications, Reid G focused on *Lactobacillus rhamnosus GR-1* and *Lactobacillus reuteri RC-14* (Liu et al., 2007; Anukam et al., 2008; Irvine et al., 2010; Hummelen et al., 2010; Hummelen et al., 2011a; Hemsworth et al., 2011; Dols et al., 2011; Hummelen et al., 2011b; Dizzell et al., 2019), using these two probiotics to intervene in women with AIDS. The reason for this may be the ability of these strains to migrate, with the most authoritative two strains colonizing the vagina: *Lactobacillus rhamnosus GR-1* and *Lactobacillus reuteri RC-14*.

Compared to developed countries, developing countries have inconsistent races, dietary habits, lifestyles, and antibiotic abuse situations (Batool et al., 2023), so research results from developed countries may not apply to developing countries. The closer ties between the institutions and countries with higher publication rankings suggested that cooperation will lead to more fruitful results. Therefore, developing countries should actively collaborate with developed countries to jointly explore the specific mechanisms of probiotics and AIDS, providing evidence for the cure or immune reconstruction of AIDS.

### 4.3 Current trends and future directions

The burst keyword map showed that future emphasis may be based on research on probiotics and vaccines. Inducing robust circulating antibody titers is a crucial goal of HIV-1 vaccination (Wilson et al., 2023).

Probiotics and their metabolites can affect the development and differentiation of bone marrow cells and B cells, as well as abnormal antibody differentiation (Wang et al., 2024). Klatt et al. (2021) investigated whether probiotic intervention could enhance the immunogenicity and protective efficacy of SIV/HIV vaccines. Their results showed that although taking probiotics can cause immunological changes, these changes do not enhance the immunogenicity of this particular SIV/HIV vaccine platform. That said whether probiotics improve vaccine efficacy may be independent of the probiotics.

However, metabolites of probiotics may affect vaccine efficacy. The probiotics used by Klatt et al. did not include Bacteroidetes, a vital source of short-chain fatty acids (propionate). Jiang et al. (2023) demonstrated that short-chain fatty acids can improve AIDS vaccine-specific immune responses, consistent with the role of short-chain fatty acids in modulating the immune system and driving antibody responses. Consequently, Huang et al. (2020) demonstrated that short-chain fatty acids can stimulate the peritoneum at high concentrations or frequent injections, leading to ascites and damage. In contrast, at low concentrations, they have no effect. Therefore, future research needs to be based on the effects of probiotic metabolites on vaccines to explore suitable probiotics to assist in vaccine enhancement.

Probiotics were not useless for vaccine development. Ninyio et al. (2024) developed a probiotic-based candidate vaccine by expressing the HIV-1 envelope membrane-proximal external region on the surface of *Escherichia coli* Nissle 1917 using CRISPR/Cas9 editing technology. This provided new ideas for the subsequent use of probiotics in the development of vaccines.

## 5 Limitations

This study only included research articles and did not include review articles, which may have affected the annual publication and citation counts. However, review articles were excluded because they only synthesize and describe previous articles, while this study required a description of future research directions. This study also used the Web of Science (WOS) Core Collection database. Incorporating different databases for format merging is a challenge in bibliometrics. However, bibliometrics generally relies on the Web of Science (WOS) Core Collection database, the most reliable citation index for scientific and academic research worldwide (Liao et al., 2024). Moreover, non-core databases generally have lower quality than Web of Science (WOS) Core Collection, so the data results are reliable.

## 6 Conclusion

This study evaluated 90 articles focused on HIV and probiotics as of May 13, 2024, using CiteSpace, VOSviewers, and Bibliometrix. Our analysis revealed an emerging field with contributions from 28 countries, 248 institutions, and 604 authors. Developing countries should actively seek cooperation with developed countries because research results from developed countries may not be suitable for populations in developing countries. It was inconclusive whether the probiotic type and its use duration affect CD4+ T-cell counts. In clinical practice, the use of probiotics for more than 30 consecutive days has been shown to have good efficacy in the treatment of AIDS-related diarrhea. Therefore, it is essential to explore the optimal strains (mixing ratios) and concentrations of probiotics for the treatment of AIDS-associated diarrhea and the improvement of clinical symptoms in patients. For future vaccine studies, researchers could start with the metabolites of probiotics and then explore suitable strains to enhance the vaccine. Using probiotics for vaccine development is also a direction that researchers could explore in the future.

## Data Availability

The original contributions presented in the study are included in the article/Supplementary material, further inquiries can be directed to the corresponding author/s.
