# Supplementary material for: Current trends and future directions in probiotics research for HIV/AIDS
Source: Front Microbiol. 2024 Dec 27;15:1444552. doi: 10.3389/fmicb.2024.1444552 (PMC11718315; doi:10.3389/fmicb.2024.1444552)
Supplement: Supplementary file 1 [file Table_1.docx]

Supplementary Material

(probiotics OR prebiotics OR synbiotics OR paraprobiotics OR postbiotics OR Probiotic)AND(hiv OR AIDS OR Immunologic Deficiency Syndrome, Acquired OR Acquired Immune Deficiency Syndrome OR Acquired Immuno-Deficiency Syndrome OR Acquired Immuno Deficiency Syndrome OR Acquired Immuno-Deficiency Syndromes OR Immuno-Deficiency Syndrome, Acquired OR Immuno-Deficiency Syndromes, Acquired OR Syndrome, Acquired Immuno-Deficiency OR Syndromes, Acquired Immuno-Deficiency OR Immunodeficiency Syndrome, Acquired OR Acquired Immunodeficiency Syndromes OR Immunodeficiency Syndromes, Acquired OR Syndrome, Acquired Immunodeficiency OR Syndromes, Acquired Immunodeficiency OR Human Immunodeficiency Virus OR Immunodeficiency Virus, Human OR Immunodeficiency Viruses, Human OR Virus, Human Immunodeficiency OR Viruses, Human Immunodeficiency OR Human Immunodeficiency Viruses OR Human T Cell Lymphotropic Virus Type III OR Human T-Cell Lymphotropic Virus Type III OR Human T-Cell Leukemia Virus Type III OR Human T Cell Leukemia Virus Type III OR LAV-HTLV-III OR Lymphadenopathy-Associated Virus OR Lymphadenopathy Associated Virus OR Lymphadenopathy-Associated Viruses OR Virus, Lymphadenopathy-Associated OR Viruses, Lymphadenopathy-Associated OR Human T Lymphotropic Virus Type III OR Human T-Lymphotropic Virus Type III OR AIDS Virus OR AIDS Viruses OR Virus, AIDS OR Viruses, AIDS OR Acquired Immune Deficiency Syndrome Virus OR Acquired Immunodeficiency Syndrome Virus OR HTLV-III)
