# Supplementary material for: Current trends and future directions in probiotics research for HIV/AIDS
Source: Front Microbiol. 2024 Dec 27;15:1444552. doi: 10.3389/fmicb.2024.1444552 (PMC11718315; doi:10.3389/fmicb.2024.1444552)
Supplement: Supplementary file 2 [file Data_Sheet_1.docx]

Supplementary Material

**supplementary figure 1** **Retrieval process flowchart for the research** 2

**supplementary figure 2 Network visualization map of journal co-citation analysis generated by VOSviewer**. 3

**supplementary figure 3 The dual-map overlay of journals in intestinal flora and HIV** 4

**supplementary figure 4 Agency outbreak words** 5

**supplementary figure 5 References outbreak** 6

**supplementary figure 6 Keywords outbreak** 7

**supplementary Table 1 Top 10 most productive journals** 8

**supplementary Table 2 Top 10 highly cited literature** 9

Publications identified through Web of Science database searching

(WoS Core Collection. SCI-Expanded)

1.Retrieval mode: Advanced Search

2.Retrieval strategy:

See attached table

3.Retrieval time: 2024.05.13

530 publications were preliminarily identified for further screening

Retrieval time span: 1995.01.01-2024.05.13

Document types: Articles

Language: (English)

Irrelevant literature was excluded by three independent authors by title, abstract and full-text screening

90 publications were included in final analysis (Online platform,Bibliometrix.VOSviewer and CiteSpace)

Institutions/Authors

Countries/Regions

Journals

Research Areas

Keywords

###### **Supplementary Figure 1：**Retrieval process flowchart for the research.


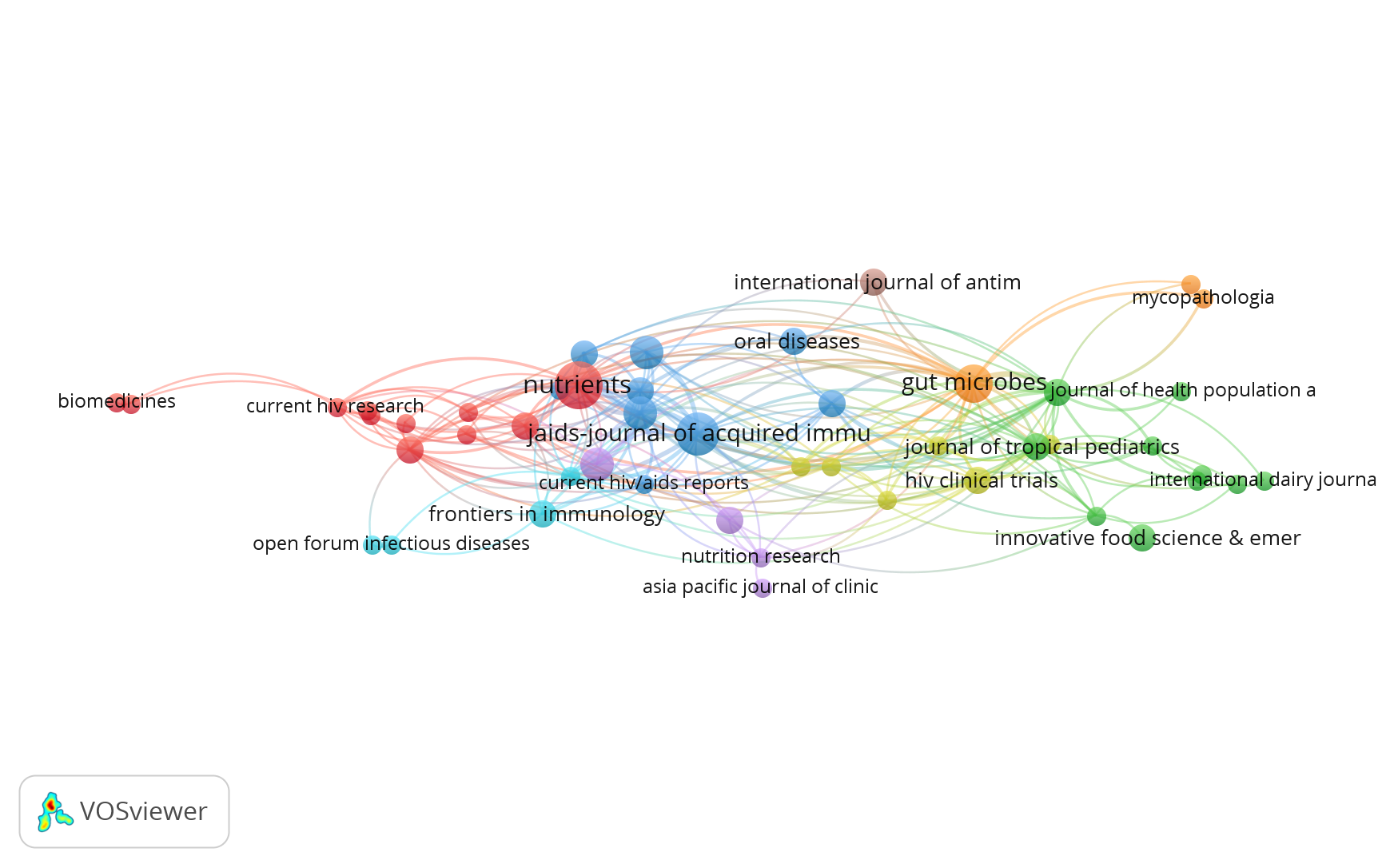


###### Supplementary Figure 2 : Network visualization map of journal co-citation analysis generated by VOSviewer.


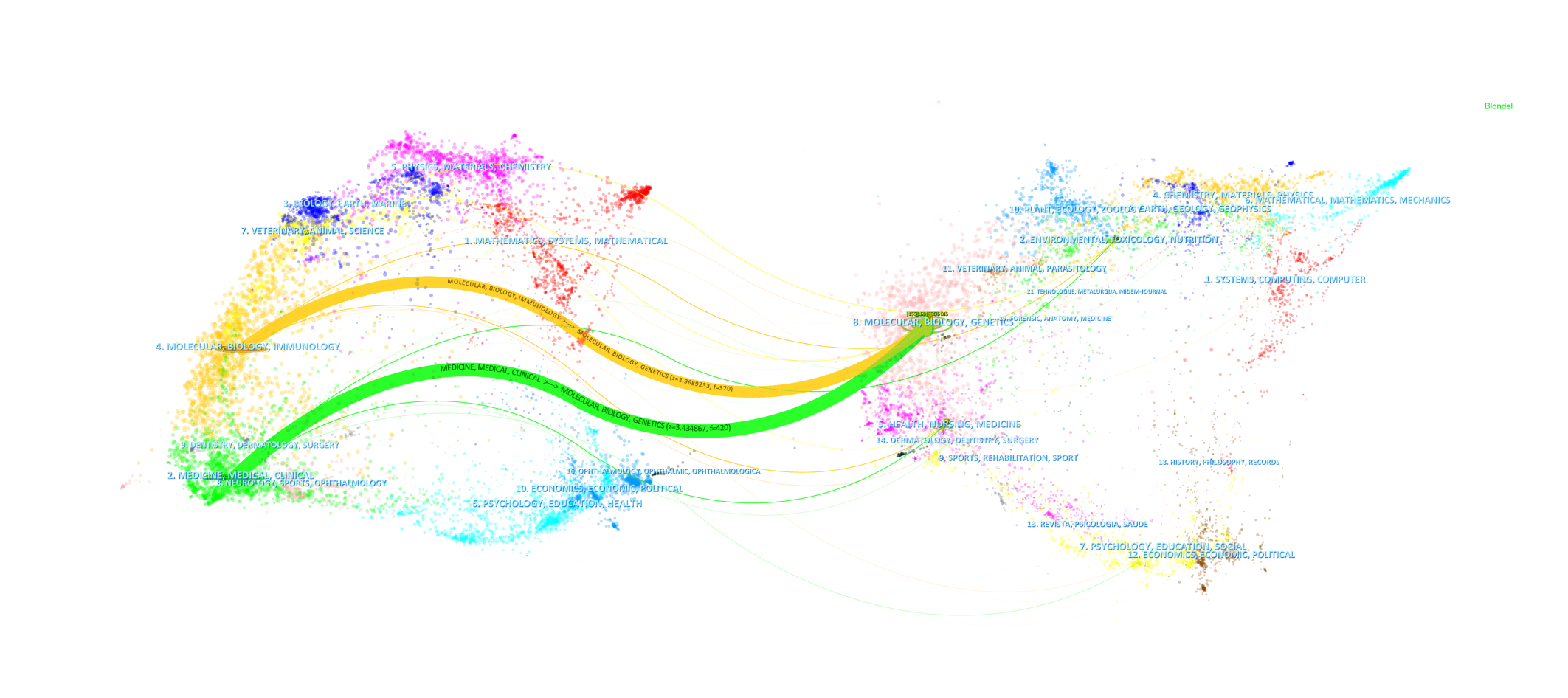


###### Supplementary Figure 3：The dual-map overlay of journals in probiotics and HIV

**Note:** A dual-map overlay of journals shows the distribution of topics. The citing journals are on the left, and the cited journals are on the right. The labels represent the disciplines covered by the journals, and the colored path represents the citation relationshi.


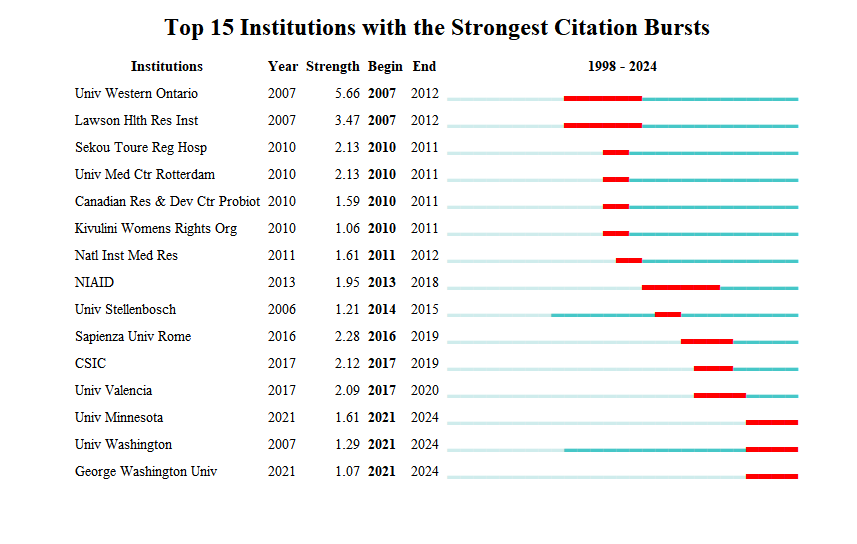


**Supplementary Figure 4：**Agency outbreak words

**Note:** When the end is highlighted in red, it signifies that the institution will be a prominent publishing organization in the future.


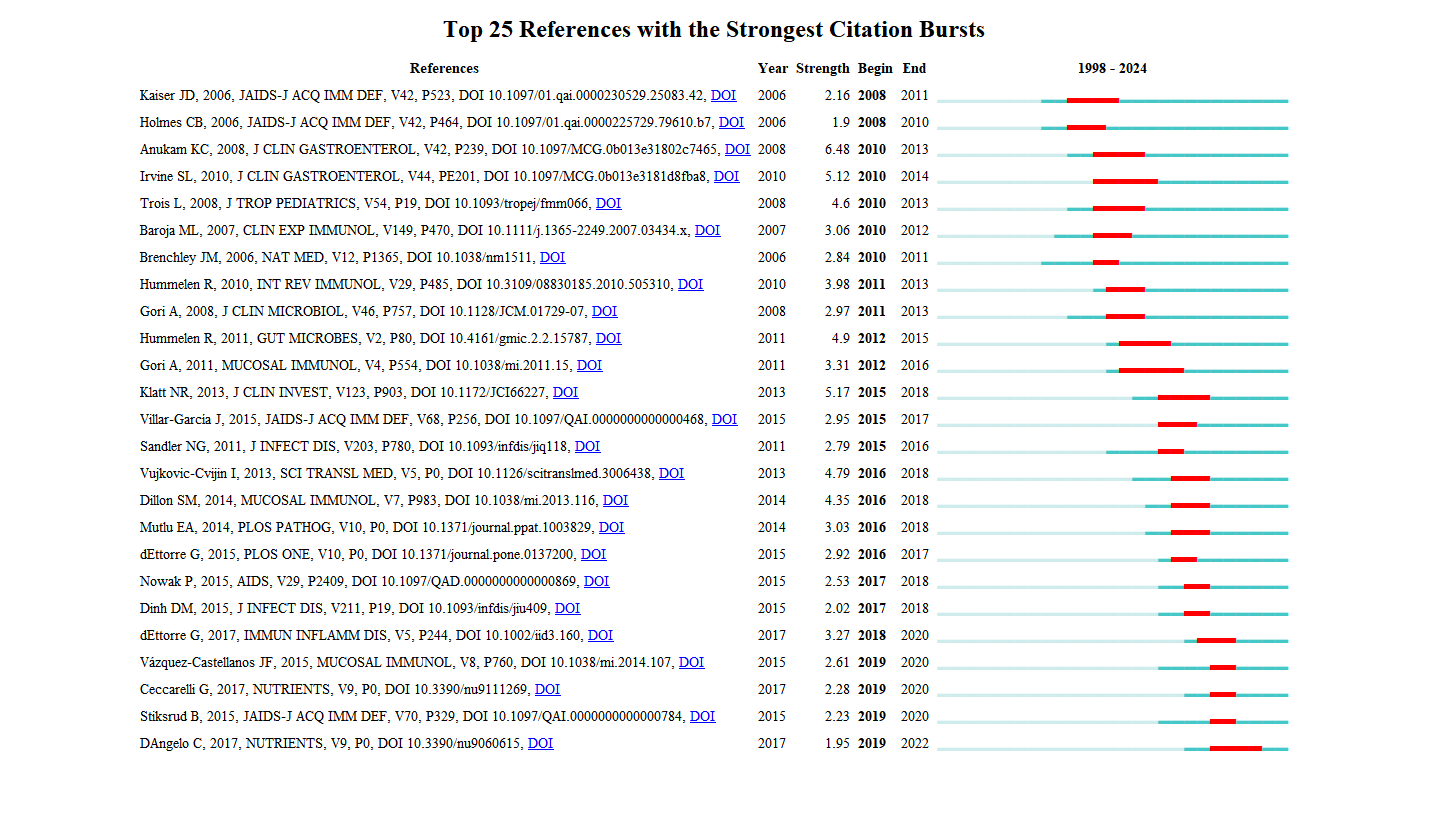


**Supplementary Figure 5：**References outbreak

**Note:** When the end is highlighted in red, it indicates that the reference is expected to be one of the top-cited references in the future.


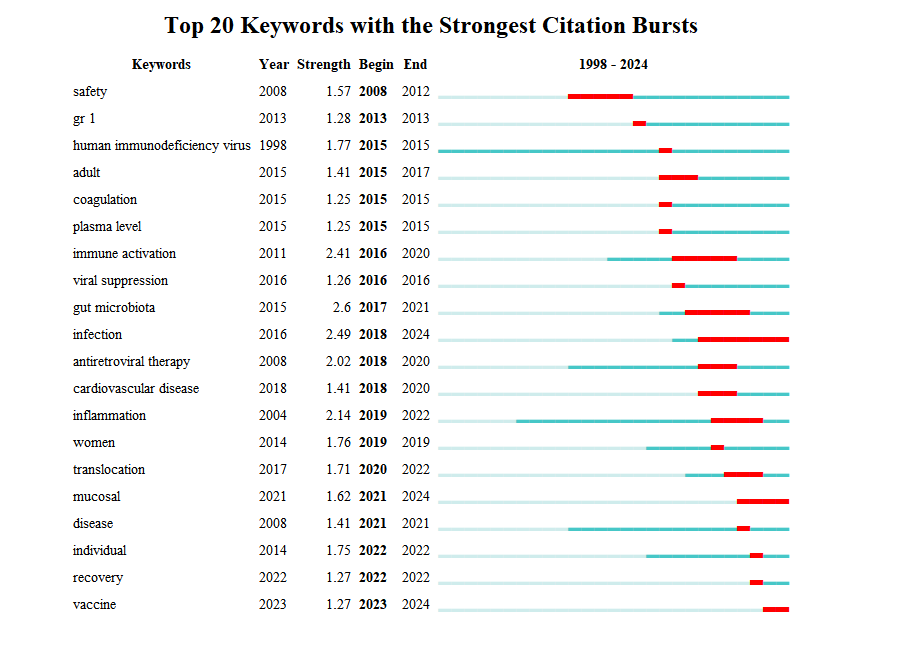


**Supplementary Figure 6：**Keywords outbreak

**Note:** The red color at the end indicates that this keyword is the main research direction for the future.

###### Supplementary Table 1：The top 10 most productive journals.

| Ranking | Journal | Output | % of 90 | IF  2023 | JCR 2023 |
| --- | --- | --- | --- | --- | --- |
| 1 | NUTRIENTS | 6 | 6.667 | 4.8 | Q1 |
| 2 | JAIDS-JOURNAL OF ACQUIRED IMMUNE DEFICIENCY SYNDROMES | 5 | 5.556 | 2.9 | Q3 |
| 3 | GUT MICROBES | 4 | 4.444 | 12.2 | Q1 |
| 4 | AIDS | 3 | 3.333 | 3.4 | Q3 |
| 5 | MUCOSAL IMMUNOLOGY | 3 | 3.333 | 8.1 | Q1 |
| 6 | SCIENTIFIC REPORTS | 3 | 3.333 | 3.8 | Q1 |
| 7 | CLINICAL INFECTIOUS DISEASES | 2 | 2.222 | 8.2 | Q1 |
| 8 | FRONTIERS IN IMMUNOLOGY | 2 | 2.222 | 5.7 | Q1 |
| 9 | HIV CLINICAL TRIALS | 2 | 2.222 | 1.821 | Q4 |
| 10 | AIDS RESEARCH AND HUMAN RETROVIRUSES | 2 | 2.222 | 1.5 | Q4 |

**Supplementary Table 2：The top 10 highly cited literature.**

| Ranking | Title | First author | Year | Citations | Citations/Year |
| --- | --- | --- | --- | --- | --- |
| 1 | Specific prebiotics modulate gut microbiota and immune activation in HAART-naive HIV-infected adults: results of the "COPA" pilot randomized trial | Gori A | 2011 | 147 | 10.50 |
| 2 | Probiotic/prebiotic supplementation of antiretrovirals improves gastrointestinal immunity in SIV-infected macaques | Klatt NR | 2013 | 128 | 10.67 |
| 3 | Gut Mucosal Barrier Dysfunction, Microbial Dysbiosis, and Their Role in HIV-1 Disease Progression | Mudd, JC | 2016 | 121 | 13.44 |
| 4 | Yogurt containing Probiotic Lactobacillus rhomnosus GR-1 and L-reuteri RC-14 helps resolve moderate diarrhea and increases CD4 count in HIV/AIDS patients | Anukam KC | 2008 | 121 | 7.12 |
| 5 | Safety and tolerance of Lactobacillus reuteri supplementation to a population infected with the human immunodeficiency virus | [Wolf BW](https://webofscience.clarivate.cn/wos/author/record/6227306) | 1998 | 108 | 4.00 |
| 6 | Probiotics Reduce Inflammation in Antiretroviral Treated, HIV-Infected Individuals: Results of the "Probio-HIV" Clinical Trial | [d'Ettorre G](https://webofscience.clarivate.cn/wos/author/record/637206) | 2015 | 106 | 10.60 |
| 7 | Probiotic Yogurt Consumption is Associated With an Increase of CD4 Count Among People Living With HIV/AIDSMicrobiome and Immune Activation Parameters in HIV Infected Subjects | Irvine SL | 2010 | 97 | 6.47 |
| 8 | Probiotics and immune response | Cunningham-Rundles S | 2000 | 97 | 3.88 |
| 9 | Effect of Probiotics (Saccharomyces boulardii) on Microbial Translocation and Inflammation in HIV-Treated Patients: A Double-Blind, Randomized, Placebo-Controlled Trial | Villar-García J | 2010 | 91 | 9.10 |
| 10 | Effect of 25 weeks probiotic supplementation on immune function of HIV patients | [Hummelen R](https://webofscience.clarivate.cn/wos/author/record/2469623) | 2011 | 85 | 6.07 |
